# “We could be good partners if we work together”: the perspectives of health and social service providers on the barriers to forming collaborative partnerships with social housing providers for older adults

**DOI:** 10.1186/s12913-022-07671-6

**Published:** 2022-03-07

**Authors:** Christine L. Sheppard, Sarah Gould, Sara J. T. Guilcher, Barbara Liu, Elizabeth Linkewich, Andrea Austen, Sander L. Hitzig

**Affiliations:** 1 St. John’s Rehab Research Program, Sunnybrook Research Institute, Toronto, Canada; 2 Department of Family and Community Medicine, University of Toronto, Toronto, Canada; 3 Leslie Dan Faculty of Pharmacy, University of Toronto, Toronto, Canada; 4 Rehabilitation Sciences Institute, University of Toronto, Toronto, Canada; 5 Regional Geriatric Program of Toronto and University of Toronto, Toronto, Canada; 6 North & East Greater Toronto Area Stroke Network, Sunnybrook Health Sciences Centre, Toronto, Canada; 7 Department of Occupational Science and Occupational Therapy, University of Toronto, Toronto, Canada; 8 Seniors Services and Long-Term Care, City of Toronto, Toronto, Canada

**Keywords:** Inter-organizational collaboration, Social housing, Access to services, Integrated care, Aging, Social care

## Abstract

**Abstract:**

**Background:**

Many older adults are aging-at-home in social housing. However, the lack of integration between housing and health services makes it difficult for older tenants to access needed supports. We examined barriers and facilitators health and social service providers face providing on-site services to older tenants.

**Methods:**

We conducted semi-structured qualitative interviews and focus groups with health and social service professionals (*n* = 58) in Toronto, Canada who provide community programs in support of older tenants who live in non-profit, rent-geared-to-income social housing. Interviews examined the barriers they faced in providing on-site services to older tenants.

**Findings:**

Service providers strongly believed that collaboration with on-site housing staff led to better health and housing outcomes for older tenants. Despite the recognized benefits of partnering with housing staff, service providers felt that their ability to work effectively in the building was dependent on the staff (particularly the superintendent) assigned to that building. They also identified other barriers that made it difficult to work collaboratively with the housing provider, including staffing challenges such as high staff turnover and confusion about staff roles, a lack of understanding among housing staff about the link between housing and health, challenges sharing confidential information across sectors, and complex and inefficient partnership processes.

**Conclusion:**

Older adult tenants are increasingly vulnerable and in need of supports but the housing provider has a long history of ineffective partnerships with service providers driven by complex and inefficient staffing models, and an organizational culture that questions the role of and need for partnerships. Findings highlight the need for more effective integration of housing and health services. Simplified processes for establishing partnerships with service agencies and more opportunities for communication and collaboration with housing staff would ensure that services are reaching the most vulnerable tenants.

## Background

Across Canada and the United States, a growing number of older adults are living in social housing [1, 2], which is a subset of affordable rental housing where rents are geared to income or supplemented with housing stipends [3]. Older adults in social housing are more vulnerable than those in the community due to high rates of social isolation, disability, food insecurity, and chronic physical and mental health conditions (e.g., [4–10]). There are also low levels of health literacy among older adults in social housing [11], contributing to heightened hospital and emergency department use [12, 13]. Given the health and social needs of older adults aging in place in social housing [2, 14], integration of care across housing, health, and social service sectors is needed.

Although different definitions of integrated care exist [15–17], Chen and Catallo ([18], p. 268) offer a perspective that emphasizes the integration of healthcare systems with social service systems (including housing), describing it as: “Health care organizations from across the continuum of care working together with social services organizations so that services are complementary and coordinated in a seamless and unified system, with care continuity for the patient/client in order to achieve desired health outcomes within a holistic perspective.” This definition highlights the need for inter-organizational collaboration arising from the fact that patients/clients require a range of services that are delivered by many organizations across sectors.

### Inter-organizational collaborations in health and social service delivery

Inter-organizational collaboration in health and social service delivery occurs when two or more organizations remain formally autonomous but form a relationship for a common goal or purpose [19]. These relationships are guided by a set of rules or structures that help to facilitate an exchange of resources, information, or services [19, 20]. Inter-organizational collaborations spanning health and social service sectors are essential for improving functional outcomes, quality of life, and quality of care, particularly for older adults with multiple health and social challenges [18, 21].

The integration of care can span five domains [15, 21]. The *funding* and *administration* domains consider government regulations and administrative functions to facilitate inter-sectoral planning, while the *organizational* domain considers the characteristics and practices of individual organizations within the collective, which may influence inter-agency planning, service affiliations, and jointly managed programs and service. The fourth domain is *service delivery*, which includes centralized client information and referrals, and case management approaches. Service delivery also affects joint training and interdisciplinary teamwork within and across organizations. The fifth domain, *clinical,* includes joint care planning, shared clinical records, common decision support tools, and ongoing communication with patients/clients. Auschra [19] recommends the inclusion of a sixth domain, *inter-organizational*, to account for the specific experiences of integrated care that occurs through inter-organizational collaboration (e.g., governance mechanisms).

Previous research has identified several enabling factors that promote health and social service integration, including organizational culture and leadership, shared vision and goals, team-based care, information-sharing and communication systems, dedicated funding and resources, and accountability agreements [22]. However, a recent systematic review identified 20 common barriers to inter-organizational collaboration spanning all domains of integrated care [19]. Barriers were most common in the *inter-organizational* and *service delivery* domains, and included lack of technology standards, varying professional standards, inefficient communication, lack of trust, lack of leadership, power imbalances, and incompatible organizational structures. This review also found that while some barriers were rooted in institutional and legal structures, many were actively and purposefully promoted by organizations working as part of the integrated partnership [19]. The author of this review concluded that more research is needed to develop a comprehensive understanding of how inter-organizational collaborations working towards integrated care are formed and developed, as well as the sources of the barriers faced during the process.

### Integrating health and social services for older adults in social housing settings

There are many examples of integrated care that have been shown to improve physical and mental health outcomes for older adult tenants in social housing (e.g., [23, 24]). One notable case comes from Ontario, Canada where community paramedics provide a health promotion program in social housing buildings with a high proportion of older adults frequently calling 911 [25]. This integrated model, called CP@Home, was shown to reduce 911 calls, improve quality of life, reduce chronic disease risk, foster linkages to primary care, and reduce financial burden on the emergency care system [4, 26, 27], highlighting the success of integrated partnerships for improving health and well-being. In this model, however, social housing is merely the location with which services are provided, and the role of the housing provider in supporting integrated care is unknown.

Other models of integrated care have tried to bridge the gaps between social housing, health, and social service sectors. This was accomplished by building infrastructure that coordinated service delivery and increased collaboration to promote access to the supports and resources that are available within the community [28–30]. For example, one model [28] used a variety of strategies to increase awareness about available community supports and build partnerships between health and housing professionals. Web-based tools (including a database of available services) increased access to resources within the community, and a centralized database helped track and share client information across agencies to identify areas of service duplication. The model was successful at bringing 30 new partnerships into the apartment building; services were more coordinated, and housing staff were able to leverage supports more effectively from their health partners through this integrated partnership [28].

In the examples described above, the integrated care models were formed through heath agencies partnering together to offer services within a social housing context; however, this research has not explored how a partnership with the social housing provider was formed, or what role the social housing provider had in supporting integrated care. For instance, it is not clear how housing policies impact the ability of health and social service agencies to provide services on-site and what role health partners feel that housing staff can play in supporting coordinated, unified, and seamless access to their services among tenants. This study used a qualitative approach to understand the experiences of health and social service providers providing services to senior tenants in social housing in Toronto, Canada. We identify key barriers and develop recommendations to enhance partnerships between service agencies and housing providers.

## Methods

This qualitative study was conducted as part of a larger study examining the needs of older adult tenants living in social housing [31]. This paper draws specifically on the experiences of health and social service providers to understand the housing-related barriers they face providing services to tenants. Doing so provides insight on what organizational factors facilitate or hinder their ability to provide services in these community settings, and what strategies they have employed to meet the health and social care needs of tenants.

### Study context

This study was conducted with a municipally owned but independently operated non-profit social housing provider located in Toronto, Canada. They operate over 350 high- and low-rise apartment buildings, including 83 buildings that are designated specifically for older adults aged 59 or older. These ‘seniors’ designated’ buildings are home to nearly 15,000 older adults who pay 30% of their annual household income towards rent (i.e., rent-geared-to-income).

Similar to other social housing providers in Ontario [2, 32], the housing provider is not funded to provide any health or social services to tenants, and thus relies on external agencies to provide these supports. Housing supports, however, are facilitated through three key staff roles: (1) superintendents and maintenance staff that maintain the buildings and units; (2) tenant services staff who manage leasing, rent calculations and payments, and rental arrears; and (3) community support staff who provide crisis support and referrals to tenants struggling to manage their tenancy. The operating model was such that staff were assigned to support multiple buildings, necessitating frequent travel between sites. The housing provider also had policies that governed exclusive and non-exclusive use of non-residential spaces by tenants and community partners. Each region was responsible for implementing these policies within local buildings, and external partners wishing to access space to facilitate programs or services were required to complete an application and provide proof of insurance to the regional housing manager.

Starting in 2016, the housing provider was working with their municipal partners to enhance services for the 15,000 older adults living in the seniors’ designated buildings. The municipality identified inconsistent and inadequate delivery of housing services to older tenants in these buildings and found a lack of integration between housing and health services. In response to these challenges, a new housing services model was developed, with three core objectives: (1) foster relationships of trust between housing staff and tenants; (2) improve the delivery of housing services, with an increased focus on issues that disproportionately impact housing stability for older tenants; and (3) increase access to health and social services through enhanced partnerships with community agencies and integration of programs directly in the building. In 2019, our research team partnered with the municipality and the housing provider to gather evidence from a variety of internal and external stakeholders to support the design and implementation of this new model [31]. The interviews described here were part of our efforts to learn more about the challenges that external health and social service agencies face supporting tenants and to develop recommendations to make it easier to provide services on-site.

### Participants

This study focuses on the experiences of health and social service professionals (*n* = 58) providing supports to older adult tenants living in social housing. Service providers were recruited through word-of-mouth and recruitment flyers shared with various municipal departments and health and social service partners known to operate in the buildings. Interested participants contacted the researcher to schedule an interview in-person (*n* = 15) or over the phone (*n* = 6). Additionally, two organizations invited frontline staff (*n* = 37) to participate in one of four pre-scheduled focus groups (two per agency). Participating service providers included geriatric psychiatrists, nursing, social workers, service navigators/coordinators, supportive housing providers, and community support service (CSS) providers working in either front-line (*n* = 48) or management (*n* = 10) roles.

### Data collection

Semi-structured interviews and focus groups explored challenges aging in place in social housing. Sample interview questions that pertained to the current study are shown in Table 1. Interviews and focus groups were approximately one hour long and were conducted by a trained interviewer (CLS). All sessions were audio-recorded and transcribed verbatim.

**Table 1:** Sample interview questions

Sample Interview Questions	
1. What are some of the key issues that impact tenants’ health and wellbeing?	
2. What factors make it easy for tenants to successfully navigate the health and social supports that are available to them? What makes it difficult to navigate these services?	
3. In your experience, how do [housing provider] staff help tenants access these services?	
4. What barriers or obstacles have you encountered providing support services to tenants on-site?	

Data was collected from November 2019 to February 2020. Ethics approval was obtained from the Research Ethics Office at Sunnybrook Health Sciences Centre (Project Identification Number 308–2019) prior to data collection. Informed consent was provided by all participants and all methods were carried out in accordance with relevant guidelines and regulations. As a thank-you for participation, focus group participants received refreshments while those doing an interview received a $10 gift card.

### Analytic approach

Our analysis drew on qualitative description, which is an approach widely used in health services research [33], as it elicits a rich description of a particular experience to inform meaningful policy and practice recommendations [34]. We followed the principles outlined by Braun and Clark [35] and Saldana [36] to identify key patterns in the data. Using methods of constant comparison, coding was completed CLS (lead researcher) and SG (research analyst with experience in qualitative research) in collaboration with the research team. Standardized techniques in qualitative research were used to establish rigour, including double-coding, maintaining an audit trail, analytic memoing, and team discussions [36]. Data management and coding was facilitated using NVivo 12.

## Results

Our analysis showed that service providers recognized benefits of partnering with housing staff; however, they felt that their ability to work effectively in the building was dependent on the housing staff (particularly the superintendent) assigned to that building. Other barriers that were identified in our analysis included staffing challenges such as high staff turnover and confusion about staff roles, a lack of understanding among housing staff about the link between housing and health, challenges sharing confidential information across sectors, and complex and inefficient partnership processes.

### Collaborative partnerships across sectors enhance outcomes for tenants

Service providers strongly believed that collaboration with on-site housing staff led to better health and housing outcomes for older tenants. In many examples, service providers felt that having a relationship with housing staff helped bring forward tenancy issues that could be supported before they became a crisis. For instance, a manager of a supportive housing program described:

*“We’ve had situations where the [tenant services staff] will reach out to us and say, ‘this person hasn’t paid their rent for two months, they’ve been a tenant of ours forever, can you work with them around this’ and many times, we’ll work with the client, find out what is going on, and then work together. We can get to the bottom [of it] … [and] it’s so much better than all of sudden, they get an eviction notice.”* (SP13, Supportive Housing Manager)

In more complex cases where older tenants were struggling with hoarding and pest control issues, service providers felt that being able to “tag team” with housing staff helped foster trust with tenants and led to greater willingness to accept supports. As one service provider described:

*“I’ve had many buildings over the years, actually, where we’ve done this, where there is hoarding and extreme clean issues. We go under a tag team and […] we can go together and discuss it. And then because they have trust in me for their health care issues, they actually realize that the superintendent isn’t out there to kick them out. Then there’s compliance. There’s more compliance […] with the plan to change.”* (SP23, Service Navigator/Coordinator)

Some service providers felt that partnerships enabled housing staff to be champions for their services, which made it easier for tenants to be aware of and willing to accept supports from community agencies. For example, one social worker who facilitated a social dining program described how the superintendents regularly encouraged tenants to go to their program and would collaborate with staff to identify tenants who might benefit from attending. A nurse practitioner had a similar experience where housing staff helped her connect with a vulnerable tenant facing chronic arrears who was previously resistant to accepting services:

*“I actually have a lady who I just saw this week, she’s been in quite chronic arrears and has been very resistant to our team working with her. So, I actually went with two housing staff, one of which she knows very well and apparently trusts. We went together and […] smoothed things over, and because of that, she let me in, and she let me sit and do my assessment, and then she let me refer her, she agreed to a referral to geriatric psychiatry [and] a couple of other things that I wanted to do for her. So that was really successful, that pairing with the housing circle.”* (SP2, Nurse Practitioner)

### Having a “good” superintendent is key to a successful partnership, but high turnover makes it difficult to form relationships

Service providers unanimously felt that a strong relationship with the superintendent was their key to success in any given building. Superintendents were viewed as ‘gatekeepers’ and it was recognized that they had intimate knowledge of the tenants and their needs because they are on-site every day. As one participant described, *“[the superintendent] is the eyes and ears [in the building]. He knows the building, he knows the tenants, he knows who is having problems, who is not having problems”* (SP19, CSS Manager).

Many service providers had examples of how they successfully partnered with the superintendent to support tenants (see Table 2). One participant remarked that theirs was even a reciprocal relationship: *“If I ask [the superintendent] something, he’s right on top of it. And he comes for help from us. My PSWs [personal support workers] speak different languages, so he taps into that for translation with tenants. So, it’s a give and take kind of thing”* (SP57, Supportive Housing Nurse).

**Table 2:** Examples of successful collaborations with the superintendent

**Collaboration**	Illustrative Quote
**Providing favours**	“Like often they’ll like help when I ask them, you know, ‘can you help to…I know this isn’t something you’re supposed to do but can you help to remove something from this unit’ or, you know.” (SP6, Social Worker)
**Facilitating access to units**	“My client was discharged from the hospital, not able to find their [substitute decision maker], so my director escalated to the supervisor in the area to get the superintendent after workhours, at 8:00 pm, to open the door for my client to be home.” (SP32, Service Navigator/Coordinator)
**Identifying tenants who need supports**	“They will come and say ‘listen, I think you need to go and take a look to 714, because I think they need some support there.’ And we will go together.” (SP54, Supportive Housing Nurse)
**Helping tenants access services**	“I had as recently as last week the super[intendent] agreed to take the person from their unit to the lobby so they could get wheel trans for hemodialysis three times a week, and ensure that once they come back, they can get back up to their unit. So that was very engaging.” (SP35, Service Navigator/Coordinator)

While a relationship with the superintendent was viewed as critical, service providers reflected that there was a high level of inconsistency across superintendents with respect to how they operate and what they view as their role, which can *“make or break what we can do the building”* (SP10, CSS Manager). While some superintendents were *“nice, very approachable and willing to do anything to help us”* (SP11, CSS Manager), others gave them a *“hard time”* and made them *“fight for what we need”* (SP12 CSS Manager), like parking spaces, access to the community room, or access to individual units.

These relationships were further strained by high staff turnover and a staffing model that required superintendents to travel between multiple buildings. This made it difficult to *“build and develop trust with [them]”* (SP1, Social Worker), as most service providers did not know who the superintendent was, what days or times they were working in a specific building, or how to reach the them when they were off-site.

The lack of presence in the buildings, coupled with *“ridiculously high”* (SP23, Service Navigator/Coordinator) turnover rates made it difficult to form relationships. As building staff shifted, working relationships suffered, especially if that new staff member replaced a superintendent they had previously worked very closely with. Others were concerned about the need to ‘socialize’ their services to the superintendent each time they shifted portfolios; many were hesitant to invest resources into building a new partnership, because they were not convinced the person would stay in the role. As one social worker described,

*“They move staff around a lot, so sometimes you get a connection with someone and you’re getting some work done and then they are moved, and it’s challenging. […] I think, sometimes I fall into the ‘oh, they’ve changed someone again’ so I think I’m also a bit hesitant, along with the tenants, to engage, because I’m not sure how long that person is going to be there, too." (SP1, Social Worker)*

For one service provider, the turnover was so excessive, they reluctantly explained that they reduced the frequency of their services in the buildings.

In addition to high turnover rates, service providers felt that the housing provider had a complex staffing model that was difficult to navigate. Outside of the superintendent, service providers were “*confused about what all the roles are”* (SP22, Service Navigator/Coordinator), and felt it was not always clear *“who to talk to, and if the person you’re talking to has any authority to help you work in a specific space […] because there is real variation in what people see as their responsibility”* (SP5, Geriatric Psychiatrist). This was particularly true when service providers were asked to support complex tenancy issues (such as non-payment of rent, pest management, or poor unit condition) that involved housing staff from different departments:

*“And then there’s the housing issues that we don’t always understand and aren’t always transparent. […] So, trying to figure out who we coordinate with and who we talk to and sometimes waiting to hear back from them forever is challenging."* (SP14, Geriatric Psychiatrist)

### Housing staff lack understanding of health Issues Impacting seniors & how services can help

There was a general recognition that the housing provider *“genuinely wants to help people”* (SP2, Nurse Practitioner) and was committed to keeping seniors housed. Many, however, were concerned that staff lacked a general understanding for how complex health issues impact tenancies, A geriatric psychiatrist described how:

*“There is a very unsophisticated approach to understanding why someone is behaving the way they are being. It’s usually related to – ‘Mrs. Jones has lived in unit 201 for thirty years, now she is collecting cats. This is bad, bad Mrs. Jones.’ – and there is an assumption that this person is capable, and they are having these behaviours for, I don’t know, some strange reason. When actually, a lot of these behaviours are the person has lost their sense of smell, they may have had a stroke, they may have medical problems, they may have psychiatric issues. I think there’s quite a lack of appreciation for what is normal aging and what are real concerns that could be supported.”* (SP5, Geriatric Psychiatrist)

Stemming from this lack of appreciation of the link between housing and health, some service providers felt that they were *“not invited”* (SP12, CSS Manager) to provide programs on-site due to a defensive *“siege mentality”* (SP5, Geriatric Psychiatrist) that questioned the need for service providers. Service providers wanted housing staff to know they are *“only there for the benefit of the people in the building”* (SP11, CSS Manager) and “*not just to give [them] a hard time and always bring issues to [them]”* (SP15, Case Manager). However, a common sentiment among service providers was that housing staff had limited understanding of who they are or how their services help tenants. Reflecting on this observation, a manager of a supportive housing program noted that:

*“So, there wasn’t a lot of understanding of our role […] but if other buildings don’t have it, and if it hasn’t been their experience and they haven’t been prepared ahead of time of this resource available to you, then they may not just see it that way. They don’t know how to utilize us or understand what we’ve been doing […] and what we’ve been able to prove works really well.* (SP14, Supportive Housing Manager)

### It is difficult to share confidential information across sectors

A major challenge faced by service providers was linked to the fact that the housing provider was *“not part of the circle of care”* (SP16, CSS Manager), and there was no reliable mechanism to facilitate the sharing of confidential information between the housing provider and service providers. The lack of access to tenancy-related information was particularly challenging for service providers, who felt they were operating in the dark because *“we don’t see every letter that [tenants] receive”* (SP3, Supportive Housing Manager). Although service providers will ask, housing staff cannot provide that information due to confidentiality. As one geriatric psychiatrist elaborated:

*“Sometimes we find out months and months and months and months later that there’s this other set of [housing] issues going on. It’s not just clutter, but there’s also this financial stuff, and they’re on a repayment schedule, which we knew nothing about. So, it’s very hard to understand: 1) how vulnerable they might be; 2) how at risk they are of losing their housing; and 3) the whole picture in terms of how they’re not managing."* (SP14, Geriatric Psychiatrist)

While some service providers had examples of times the housing provider reached out to get support for a housing-related issue, most relied on informal consent mechanisms, asking the client for permission to liaise with housing on a case-by-case basis. A manager at a community support service agency discussed that this sometimes limited how much support they were able to provide to tenants:

*“We can only support as far as the tenant will consent to. If there is a serious tenancy issue, and the tenant says, ‘I don’t want you to talk to them’, that can certainly limit what we can do […] to support that individual*." (SP10, CSS Manager)

While some service providers felt it was easy to *“just get consent”* (SP16, CSS Manager) because tenants had trust in them as health care providers, others noted that tenants *“often say no a lot”* (SP1, Social Worker) and preferred to keep their health *‘separate’* from their housing. Although service providers understood this position, it was also recognized that keeping housing and health services siloed made it more difficult to help tenants.

The fact that housing staff *“are not health care providers*” (SP14, Geriatric Psychiatrist) and thus not part of the circle of care also created privacy challenges, particularly when housing made referrals for services. One nurse practitioner described her strategy for managing this:

*It’s sort of a bit of a fine line because there’s confidentiality, so I’m not going to someone and saying, ‘on, well they’ve got all these things wrong with them.’ But I will say that we try to loop back – especially if [the housing provider] referred the person, we try to look back to [housing] and say, ‘you know, look, our team has been in, we’re assessing and we’re working on these issues with that person, so hopefully things will get better.”* (SP2, Nurse Practitioner)

### Complex and inefficient partnership processes hinder the provision of on-site services

It was clear from the interviews that the housing provider’s process for establishing formal partnerships with external partners was inconsistent across buildings and filled with red tape. Service providers cited challenges in formalizing their partnership, and difficulties relying on that partnership to carry out their services, which they felt made housing an unreliable partner.

Although some participants were able to provide supports to tenants without establishing any formal partnership, others described extensive paperwork that they were required to complete. There was also no consistency with the types of documents that service providers were asked to provide or what stipulations were included in the partnership agreement. For example, one social service agency that provided a variety of health and wellness services in multiple buildings pointed out that they were required to pay rent in some, but not all, of their sites. The manager of this program described the stress this lack of consistency created:

*“We’re always worried that one day [the housing provider] is going to say, ‘Oh, we’re going to start charging [rent for] the others when we always wonder why do we even pay rent in the first [place]? I’m in the middle of negotiating the next lease and they’re raising the rent, raising the rent, raising the rent. […] It just doesn’t make sense at all. We could pull out, right? What if we didn’t have fitness classes, lunch programs, farmer’s markets, supportive housing? So then where are the tenants going to be? It’s so idiotic. It’s very frustrating."* (SP10, CSS Manager)

Conversely, another service provider who had been running a community program in numerous buildings discussed how their partnership underwent a recent shift from an informal handshake agreement to something more “formal” and “legitimate,” which fostered a greater sense of security for the program:

*"[This year], we filled out a lot more forms to secure the space and make things more legitimate. Before we just kind of walked in, they were like ‘yah! Come on in!’ like, super loose goose. But now it’s become more formal. […] We show that we have insurance, we have police checks, we’re not selling anything. All these different things that we had to check off. […] They wanted to see all our financials. […] Now we have that security.”* (SP11, CSS Manager)

In some cases, the paperwork was thought to be both inappropriate and difficult to complete, which left many service providers confused about why they had to jump through so many hoops to provide a service to tenants. For instance, one nurse practitioner trying to establish a new clinic in a building described how their paperwork took six months to finish, which delayed the start of her program:

*[The housing provider] had this massive, and really, I thought, difficult to negotiate contract that had to be signed by my health organization. […] they wanted all these details about where my program got funded from and they wanted the funding agreements. I thought it was a bit much. Not just invasive, but difficult, right? Why do you need to see the funding agreement of how I get paid? So that took a little while to get completed because it was such an onerous document and it had to go to senior management of my organization. […] I felt a bit funny because it’s like, I’m offering you this nurse practitioner clinic. Why are you not jumping all over this?* (SP2, Nurse Practitioner)

Service providers also found that the person-to-person negotiations with staff in the buildings often created unnecessary roadblocks to delivering programs. For example, several providers had difficulties accessing community rooms to meet with clients while others received parking tickets for parking their car in the building parking lot. Service providers were bewildered about why they would face any resistance from the housing provider at all, given that the supports they are offering are of no cost to the housing provider and are only there to benefit the tenants. This resistance, be it in the form of a non-response from housing staff or additional hoops to jump through, created a negative environment that made it difficult for service providers to *want* to provide programs in those spaces. As one service provider described:

*We’re always having to frame it in a way, it costs nothing to them. They’re giving us the party room for free. We’re only benefiting the people in your building. We’re only there for a positive. I don’t know why [we get] pushback, like why are you fighting us on this? We’re only there to support you.* (SP10, CSS Manager)

## Discussion

Despite the perceived importance of inter-organizational collaborations with the housing provider, service providers faced barriers establishing successful partnerships and experienced that some buildings were easier to work in than others, depending on which housing staff were assigned to the building. While some barriers stemmed from institutional structures and system-level challenges, others were more actively raised by the behaviours of housing staff working on-site. Findings point to several opportunities for social housing providers to implement new policies and practices that foster more effective partnerships with health and social service agencies.

In the current study, service providers reported a great deal of frustration at the way the housing provider fostered partnerships that created unnecessary delays to providing services. Frustration with the overall approach to partnerships was compounded by confusion about housing staff roles and responsibilities, as well as uncertainty about who has decision-making authority. These types of inter-organizational leadership challenges have also been documented in other research on integrated care [19], and studies show that coordinated leadership efforts are essential for reducing bureaucracy and demonstrating commitment to the partnership [22, 37, 38].

While formalizing partnerships with the housing provider was challenging, service providers also faced barriers developing relationships of trust and respect with the superintendent. Prior research shows that divergent schedules and work assignments, as well as inconsistent views about roles can impede inter-organizational collaboration [19]; in the current study, these incompatible organisational processes hindered communication between partners and fostered a sense of futility among service providers, who were weary of investing resources into a relationship with a superintendent because they were unlikely to stick around. Service providers were also sometimes unsure of what role the superintendent could play on the integrated care team, observing that some were more open to collaboration than others. This potential misalignment of expectations may erode the success of integrated partnerships [37, 39]. Formal partnership agreements that outline role expectations may be one strategy to mitigate this challenge. However, having other tenant-facing support staff present in the buildings may also shift in the role of the superintendent within integrated partnerships [40]. For instance, the new housing services model being implemented in the seniors’ designated buildings will create a new role called the 'Senior Services Coordinator.' This person will be assigned to one or two buildings and will work directly on-site to build relationships of trust with tenants. Based on prior research of similar roles, this staff person will likely be a key partner for health and social service providers as they can help identify tenants who have unmet needs and foster linkages to appropriate services [40, 41]; future research will need to explore their role within the integrated care team.

In addition to a challenging staff model, collaboration was further eroded by a perceived lack of *mutual understanding* around the need for and role of integrated partnerships with health and social service agencies. Prior research shows that a common understanding of integrated care as a concept is critical for success [22]; however, when one partner has little understanding of the goals or behaviours of the other [19] and the benefits of integrated care are not mutually understood [37], interorganizational collaborations are hampered. In the current study, service providers sensed that housing staff lacked an appreciation for how complex health issues negatively impact tenancies and failed to understand how their services help tenants. Mutual understanding was further hampered by the complexity of the housing system and lack of clarity around who should be consulted to support housing-related vulnerabilities (e.g., tenancy management, pest control, repairs).

Stemming from a lack of shared understanding of roles and common goals, service providers perceived that some superintendents were *resistant* to partnerships*.* Prior research has shown that commitment to and belief in the integrated care model is key for achieving positive outcomes [38], but unwillingness to engage in interorganizational collaboration is common when individuals feel as though the partnership was forced upon them [37]. Therefore, it could be that superintendents are not being given the resources that they need to fully understand the role of on-site health and social services, leading to an unwillingness or inability to support the integrated team.

Consistent with prior research, confidentiality emerged as a key factor that limited how much support service providers were able to give [19, 22]. While this challenge predominately existed because of the siloed nature of housing and health, service providers and housing partners also had no secure information technology platform to share information in the cases where tenants provided consent. This lack of infrastructure is a known barrier [37] and research suggests that successful inter-organizational collaboration requires information sharing systems to enhance communication and information flow across partners [42, 43].

### Recommendations for practice

Older adult tenants are increasingly vulnerable and in need of supports but the housing provider has a long history of ineffective partnerships with service providers driven by complex and inefficient staffing models, and an organizational culture that questions the role of and need for partnerships. Many of the challenges described by service providers can be mapped to the inner setting of the housing provider [44], suggesting that there are opportunities for housing to implement strategies to mitigate these challenges. As such, we present four key recommendations to promote more effective partnerships between the housing provider and health and social service agencies: (1) standardized partnership frameworks; (2) proactive consent processes; (3) joint team meetings and training; and (4) on-site resources.

#### Standardized partnership framework

To facilitate more effective partnerships, a standardized partnership framework is needed. This would include a process for establishing roles, shared goals, and responsibilities and accountabilities between the housing provider and service providers. Findings from the current study stress the importance of a consistent partnership approach across buildings and to finding ways to simplify red tape by ensuring the paperwork is not onerous.

#### Proactive consent processes

While a shared record keeping system for housing and health partners is unlikely, a simplified proactive consent process would allow the housing provider to safely share relevant tenancy information with service providers working on-site. This builds off best practices observed in other jurisdictions [32], which recommend plain language consent forms that explain how their housing information will be shared and the benefits of doing so, as well as having regular opportunities to review consent.

#### Joint team meetings and training

There is a clear need for joint team meetings between housing staff and service providers. These meetings would provide opportunities to discuss high-risk tenants and explore how partners can come together to support their tenancy. These meetings would also facilitate the establishment of a shared vision, trust, and mutual understanding of how partners can work together, which will promote a more successful inter-organizational collaboration [19, 42]. There are also opportunities for more training to help housing staff understand age-related health challenges, how they impact tenancies, and the types of services are available to support those challenges. Such training may make it easier for housing staff to understand and appreciate the benefit of inter-organizational partnerships, facilitating greater openness to collaboration with health and social service agencies [37, 38].

#### On-site resources

Service providers identified several resources that they felt would make it easier for them to physically be on-site. This included touch-down spaces to meet with clients or complete work in-between client meetings as well as designated parking spaces. A publicly posted repository of housing staff (including their role, building office hours, and contact information) would also help service providers connect with housing staff, which is particularly important for buildings with high rates of turnover.

### Limitations

This study presented an in-depth perspective from service providers working across multiple buildings and in a variety of roles. However, there are some limitations which must be considered. First, we did not explore the experiences of older tenants who participate in the health and social services offered by external partners. They have their own perspectives on their service needs and the barriers they face accessing these services that should be explored in future research. Secondly, our study did not explore partnership-related challenges from the perspective of the housing provider. For instance, our sample did not include superintendents who were viewed as an integral member of the collaboration. Furthermore, our data identified numerous challenges related to housing staff turnover and accessibility of staff on-site that will be critical to address to enhance integrated partnerships. Therefore, additional research with the housing provider is needed to understand the root of these issues and develop strategies to strengthen staffing models. Such research may also uncover additional barriers that impede meaningful collaboration with health and social service agencies from the perspective of housing staff. As the new housing services model is implemented in the seniors’ designated buildings, it will also be important to examine how our recommendations have been implemented, as well as to understand the role of the new seniors’ services coordinator as a member of the integrated care team.

## Conclusions

Older adults living in social housing communities face a variety of challenges that negatively impact their ability to age-in-place. While there exist health and social services to support these vulnerabilities, service providers identified several barriers to establishing effective inter-organizational partnerships with the housing provider. As such, our findings point to opportunities to strengthen integrated partnerships by reducing bureaucratic red tape and facilitating more opportunities for communication and collaboration, which would ensure that services are reaching the most vulnerable tenants.

## Data Availability

The data that support the findings of this study are available from the corresponding author upon reasonable request.
